# CHST1 Drives Immunotherapy Resistance in Triple‐Negative Breast Cancer by Orchestrating an Immunosuppressive Microenvironment via the NKRF–CCL20–Macrophage Axis

**DOI:** 10.1002/advs.77356

**Published:** 2026-08-24

**Authors:** Shu‐Hao Jiang, Xiao‐Han Ying, Ruo‐Qing Wang, Qun Zhang, Li Chen, Zhi‐Ming Shao, Jun‐Jie Li

**Affiliations:** ^1^ Department of Breast Surgery Key Laboratory of Breast Cancer in Shanghai Fudan University Shanghai Cancer Center Shanghai China; ^2^ Department of Oncology Shanghai Medical College Fudan University Shanghai China

**Keywords:** CCL20, CHST1, immunotherapy resistance, NKRF, noncanonical signaling, triple‐negative breast cancer, tumor immune microenvironment

## Abstract

Triple‐negative breast cancer (TNBC) derives limited benefit from immune checkpoint blockade, largely owing to primary resistance shaped by the tumor immune microenvironment. Here, baseline transcriptomic profiling of a neoadjuvant chemoimmunotherapy cohort identified carbohydrate sulfotransferase 1 (CHST1) as a candidate resistance‐associated gene. High CHST1 expression was associated with poor immunotherapy response and inferior clinical outcome across clinical datasets. Functionally, CHST1/Chst1 had minimal effects on tumor‐cell‐intrinsic proliferation, migration, or invasion in vitro, but promoted tumor growth in an immune context–dependent manner in vivo. Mechanistic analyses support a model in which CHST1 associates with NF‐κB‐repressing factor (NKRF) and limits its nuclear accumulation, a localization change associated with increased NF‐κB‐related CCL20 expression. Tumor‐cell CCL20 perturbation and rescue experiments further supported a functional contribution of CCL20 to macrophage recruitment and M2‐like remodeling. Pharmacologic CCR6 blockade enhanced the antitumor activity of anti–PD‐1 therapy in syngeneic TNBC models. Finally, a CHST1–NKRF–CCL20 expression signature showed potential value for stratifying immunotherapy response. Collectively, this study supports a CHST1–NKRF–CCL20 regulatory model and identifies CCR6 blockade as a potential therapeutic strategy.

## Graphical abstract

Proposed model of CHST1‐associated immune remodeling in triple‐negative breast cancer. In CHST1‐low tumors, greater nuclear accumulation of NKRF is associated with repression of an NF‐κB‐related CCL20 transcriptional program and an immune‐inflamed microenvironment. In CHST1‐high tumors, CHST1 associates with NKRF and limits its nuclear accumulation, contributing to increased CCL20 expression, M2‐like macrophage enrichment, reduced cytotoxic T‐cell infiltration, and an immune‐suppressive tumor microenvironment.

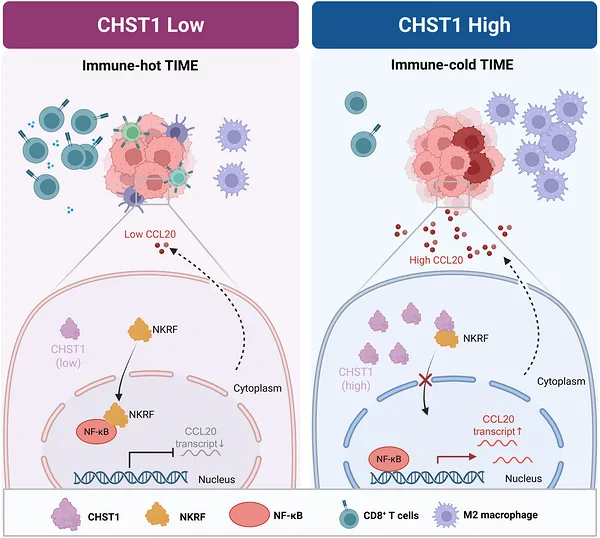

## 1 Introduction

Breast cancer remains a major global health burden among women. A recent Global Burden of Disease 2021‐based analysis reported that breast cancer accounted for approximately 2.08 million incident cases, 0.66 million deaths, and 20.25 million disability‐adjusted life years worldwide in 2021, representing the highest burden among major female‐specific cancers [1]. Among breast cancer subtypes, triple‐negative breast cancer (TNBC) stands out as a major clinical challenge owing to its aggressive behavior, pronounced heterogeneity, and the unfavorable outcomes seen in a substantial proportion of patients [2, 3]. In recent years, immune checkpoint inhibitors (ICIs), particularly PD‐1/PD‐L1 blockade, have expanded therapeutic options for TNBC. However, the overall response rate remains modest, and a considerable fraction of patients exhibit primary resistance [4, 5]. Existing biomarkers—including PD‐L1 expression—provide limited predictive value across treatment contexts [6, 7]. Therefore, there is an urgent need not only to elucidate the mechanisms governing ICI responsiveness but also to identify robust, actionable vulnerabilities that can be targeted to overcome therapeutic resistance.

Accumulating evidence indicates that immunotherapy efficacy is not solely dictated by tumor‐intrinsic features but is profoundly shaped by the tumor immune microenvironment (TIME) [8, 9]. The TIME is often conceptualized along a spectrum ranging from immune‐inflamed (“hot”) to immune‐desert/excluded (“cold”) phenotypes, which broadly correlate with sensitivity to ICIs [10, 11]. Nevertheless, clinical observations consistently demonstrate that even tumors with apparent immune infiltration can fail to respond, suggesting that the mere presence of tumor‐infiltrating immune cells is insufficient; rather, their functional states and composition often dictate therapeutic outcomes [12, 13]. In particular, myeloid cells—especially tumor‐associated macrophages (TAMs)—are major architects of immune suppression, capable of dampening cytotoxic T‐cell function and limiting responses to checkpoint blockade [14, 15]. While tumor‐derived cytokines and chemokines are known to mediate macrophage recruitment and polarization, the upstream molecular “switches” within tumor cells that trigger this immunosuppressive program remain poorly defined.

To address this gap, we analyzed baseline transcriptomes from a TNBC neoadjuvant immunotherapy cohort and identified carbohydrate sulfotransferase 1 (CHST1) as a candidate resistance‐associated gene. Distinct from its canonical role in keratan sulfate biosynthesis, we show that CHST1 promotes immune suppression through a non‐canonical mechanism that is largely uncoupled from its canonical sulfotransferase activity. Specifically, our findings support a model in which CHST1 associates with NKRF and restricts its nuclear accumulation, which may reduce NKRF‐mediated repression of an NF‐κB‐associated CCL20 transcriptional program. Tumor‐cell CCL20 perturbation and rescue experiments further suggest that CCL20 contributes to macrophage recruitment and M2‐like remodeling. Finally, pharmacologic CCR6 blockade improved TNBC responsiveness to anti–PD‐1 therapy in syngeneic models.

## 2 Results

### 2.1 Identification of CHST1 Associated with Immunotherapy Resistance in the FASCINATE‐N Neoadjuvant Immunotherapy Cohort

**FIGURE 1:**
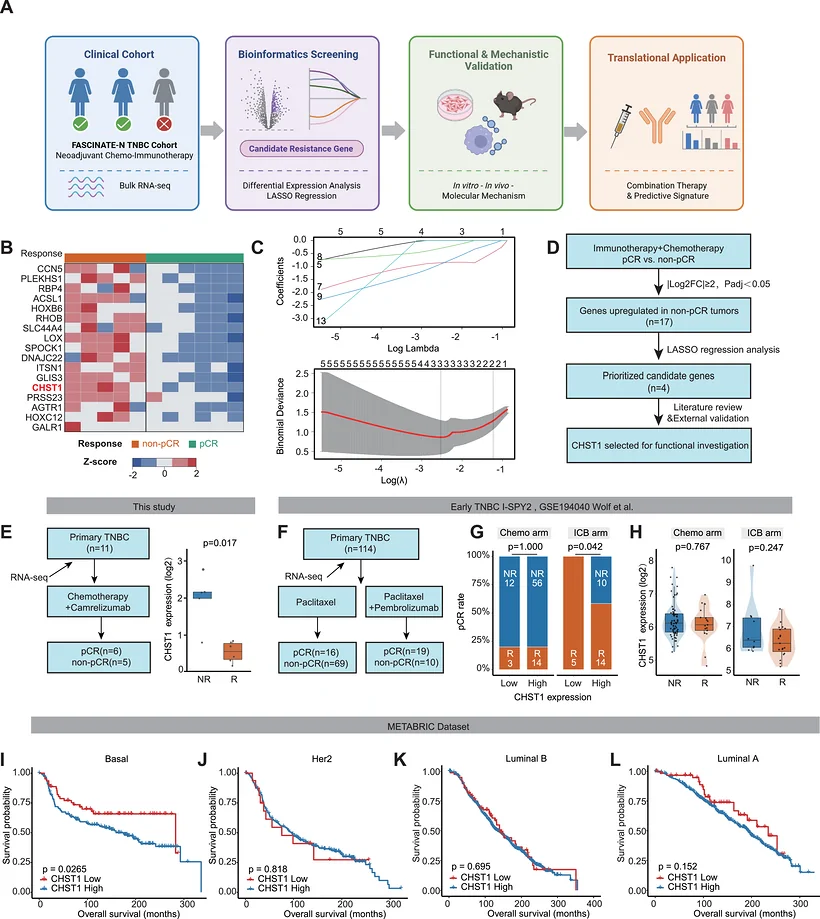
CHST1 is prioritized as an immunotherapy resistance‐associated candidate gene in TNBC and is associated with poor clinical outcome. (A) Schematic overview of the study workflow, including the clinical discovery cohort, transcriptomic screening, functional and mechanistic validation, and translational evaluation. (B) Heatmap showing the expression patterns of genes significantly upregulated in non‐pCR tumors from baseline RNA‐seq analysis of the FASCINATE‐N TNBC neoadjuvant chemoimmunotherapy cohort. Expression values were Z‐score normalized by gene across samples. (C) LASSO regression analysis was performed on genes upregulated in non‐pCR tumors to prioritize candidate genes associated with poor response to neoadjuvant chemoimmunotherapy. (D) Schematic workflow of the integrated candidate‐gene prioritization strategy, including differential expression analysis, LASSO‐based selection, external validation, and literature review, which led to the selection of CHST1 for subsequent functional and mechanistic investigation. (E) Overview of the FASCINATE‐N discovery cohort and comparison of CHST1 expression between responders and non‐responders in baseline tumor samples. (F) Overview of the I‐SPY2 early TNBC cohort, including patients treated with paclitaxel alone or paclitaxel plus pembrolizumab. (G) pCR rates in the I‐SPY2 cohort stratified by CHST1 expression in the chemotherapy‐only and chemoimmunotherapy arms. (H) Comparison of CHST1 expression between responders and non‐responders in the chemotherapy‐only and chemoimmunotherapy arms of the I‐SPY2 cohort. (I–L) Kaplan–Meier analysis of overall survival in METABRIC breast cancer subtypes, including basal‐like, HER2‐enriched, luminal B, and luminal A tumors, stratified by CHST1 expression.

To uncover the potential molecular mechanisms influencing immunotherapy efficacy in TNBC, we conducted a retrospective analysis of baseline transcriptomic data from 11 patients in our FASCINATE‐N cohort (Figure 1A). The study design and clinical outcomes of this cohort have been previously reported [16]. Briefly, all patients received neoadjuvant chemoimmunotherapy and were stratified into pathological complete response (pCR) and non‐pCR groups based on postoperative evaluation (Table S1). Differential gene expression analysis revealed distinct transcriptomic profiles between responders and non‐responders, identifying 17 genes that were significantly upregulated in the non‐pCR group (Figure 1B). To further prioritize candidate genes associated with poor immunotherapy response, we performed LASSO regression analysis on genes upregulated in non‐pCR tumors (Figure 1C). *CHST1* was retained in the LASSO model and was selected for further investigation based on its consistent upregulation in non‐pCR samples, validation signals in external datasets, and limited prior mechanistic characterization in breast cancer immune regulation (Figure 1D). Therefore, *CHST1* was prioritized as a candidate immunotherapy‐resistance‐associated gene for subsequent functional and mechanistic studies.

We next evaluated the clinical relevance of *CHST1* in both our internal cohort and the external I‐SPY2 neoadjuvant cohort. High *CHST1* expression was associated with inferior response to immunotherapy‐containing treatment, whereas this association was not observed in the chemotherapy‐only group, suggesting that *CHST1* may be more closely linked to immune‐dependent therapeutic resistance than to general chemotherapy resistance (Figure 1E–H). In the FUSCC TNBC dataset, *CHST1* expression was significantly higher in tumor tissues than in adjacent normal tissues (Figure S1A). Moreover, analysis of the METABRIC cohort showed that elevated *CHST1* expression was associated with worse overall survival specifically in the basal‐like subtype (Figure 1I–L). Together, these clinical and transcriptomic observations supported the prioritization of *CHST1* for subsequent functional and mechanistic investigation.

### 2.2 The Tumor‐Promoting Effect of CHST1 Is Immune Microenvironment‐Dependent

To define the biological role of *CHST1* in TNBC, we first examined basal *CHST1/Chst1* expression across a panel of human TNBC and murine mammary tumor cell lines (Figure S2A–D). Based on endogenous expression levels, the intended direction of genetic perturbation, and experimental context, MDA‐MB‐468 and 4T1 cells were selected for loss‐of‐function studies, whereas MDA‐MB‐231 and AT3 cells were used for gain‐of‐function studies. This design allowed us to evaluate CHST1 function in both human TNBC models and murine syngeneic systems suitable for immune‐competent in vivo experiments. Efficient CHST1/Chst1 depletion or overexpression was confirmed before subsequent functional assays (Figure S2E–L). Notably, modulation of CHST1/Chst1 expression did not markedly affect cell‐autonomous proliferation, as assessed by colony formation and CCK‐8 assays, in either human or murine TNBC models (Figure S2M–X). Consistently, Transwell assays showed no significant changes in migratory or invasive capacities following CHST1/Chst1 manipulation (Figure S3A–F).

**FIGURE 2:**
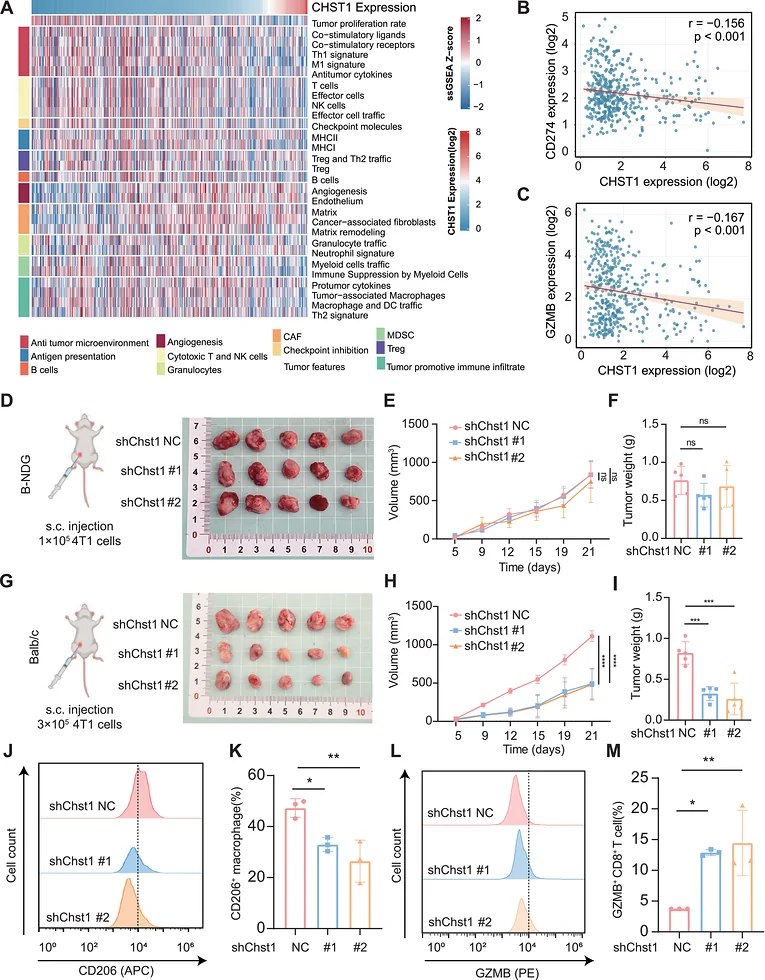
Chst1 knockdown suppresses tumor growth in an immune microenvironment‐dependent manner and remodels tumor‐infiltrating immune cells. (A) Heatmap of tumor microenvironment‐related ssGSEA scores across TNBC tumors ordered by CHST1 expression. (B, C) Correlations between CHST1 expression and CD274 (B) or GZMB (C) expression in the FUSCC TNBC cohort. (D) Experimental schematic and representative endpoint tumor images from immunodeficient B‐NDG mice injected with 4T1 cells stably expressing shChst1 NC, shChst1#1, or shChst1#2. (E, F) Tumor growth curves (E) and endpoint tumor weights (F) of 4T1‐shChst1 NC, 4T1‐shChst1#1, and 4T1‐shChst1#2 tumors in immunodeficient B‐NDG mice. (G) Experimental schematic and representative endpoint tumor images from immunocompetent BALB/c mice injected with 4T1 cells stably expressing shChst1 NC, shChst1#1, or shChst1#2. (H, I) Tumor growth curves (H) and endpoint tumor weights (I) of 4T1‐shChst1 NC, 4T1‐shChst1#1, and 4T1‐shChst1#2 tumors in immunocompetent BALB/c mice. (J, K) Representative flow cytometry histograms of CD206 expression in tumor‐infiltrating macrophages (J) and quantification of CD206^+^ macrophages among tumor‐infiltrating macrophages (K) in the indicated Chst1 knockdown groups. (L, M) Representative flow cytometry histograms of Granzyme B expression in tumor‐infiltrating CD8^+^ T cells (L) and quantification of Granzyme B^+^ CD8^+^ T cells (M) in the indicated Chst1 knockdown groups. Data are presented as mean ± SEM. Statistical significance was determined as described in the Methods. ns, not significant; ^*^ *p* < 0.05; ^**^ *p* < 0.01; ^***^ *p* < 0.001; ^****^ *p* < 0.0001.

Given the limited cell‐intrinsic effects of CHST1/Chst1 manipulation in vitro, we next asked whether CHST1 might promote tumor progression through remodeling of the tumor microenvironment (TME). Analysis of public TNBC transcriptomic datasets showed that CHST1‐high tumors exhibited reduced enrichment of multiple antitumor immune signatures, including T‐cell/effector‐cell activity, NK‐cell activity, antigen‐presentation, and MHC‐related programs, consistent with a relatively immune‐cold TME profile (Figure 2A). In line with this observation, CHST1 expression was inversely correlated with immune activity–associated genes, including CD274 and the cytotoxic effector marker GZMB (Figure 2B,C and Figure S3G–J).

To test whether the tumor‐promoting effect of CHST1 depends on an intact immune microenvironment, we compared tumor growth in immunodeficient and immunocompetent murine models. Consistent with the lack of major cell‐autonomous effects in vitro, Chst1 knockdown produced only minimal growth inhibition in immunodeficient mice (Figure 2D–F). In contrast, the same Chst1 knockdown markedly suppressed tumor progression in immunocompetent hosts (Figure 2G–I). This differential effect suggests that CHST1/Chst1 promotes tumor growth primarily through immune‐context‐dependent mechanisms rather than by directly enhancing tumor‐cell proliferation. Flow cytometric analysis further showed that Chst1 knockdown reduced CD206^+^ macrophage accumulation and increased Granzyme B^+^ CD8^+^ T cells within tumors (Figure 2J–M), supporting a role for CHST1 in shaping an immunosuppressive TME.

Together, these findings indicate that the tumor‐promoting function of CHST1 is largely dependent on an intact immune system and is associated with TME remodeling toward an M2‐like macrophage‐enriched and cytotoxic T‐cell‐impaired state.

### 2.3 Tumor Cell CHST1 Expression Relates to M2 Macrophage Marker Profile

**FIGURE 3:**
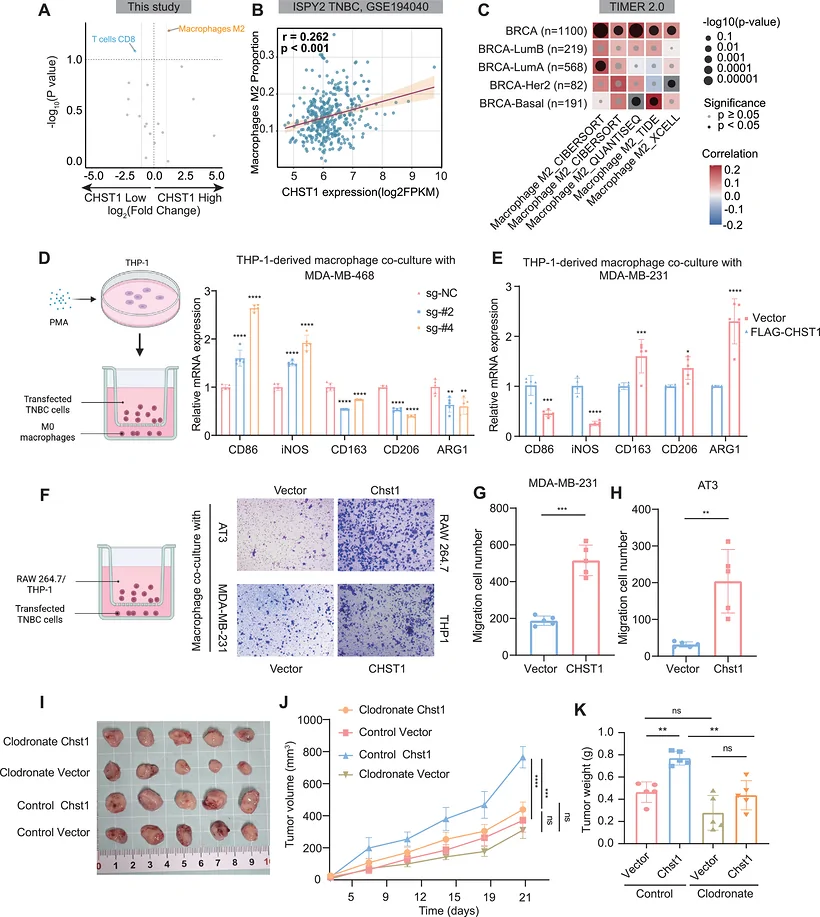
CHST1 is associated with M2‐like macrophage enrichment and promotes macrophage‐mediated tumor progression. (A) Differential abundance analysis of tumor microenvironment cell populations between CHST1‐high and CHST1‐low tumors in this cohort. Immune‐cell proportions were estimated using CIBERSORTx, and representative immune populations are highlighted. (B) Correlation between CHST1 expression and the estimated proportion of M2 macrophages in the I‐SPY2 TNBC cohort. (C) Correlation heatmap showing the association between CHST1 expression and macrophage‐related infiltration estimates across breast cancer subtypes using TIMER 2.0. (D) Schematic of the non‐contact co‐culture system and RT‐qPCR analysis of macrophage polarization markers in PMA‐differentiated THP‐1‐derived macrophages co‐cultured with CHST1‐depleted MDA‐MB‐468 cells. (E) RT‐qPCR analysis of macrophage polarization markers in PMA‐differentiated THP‐1‐derived macrophages co‐cultured with CHST1‐overexpressing MDA‐MB‐231 cells. (F) Representative images from Transwell macrophage recruitment assays using conditioned or co‐culture systems with CHST1/Chst1‐overexpressing TNBC cells. (G, H) Quantification of migrated macrophages in Transwell recruitment assays using CHST1‐overexpressing MDA‐MB‐231 cells (G) or Chst1‐overexpressing AT3 cells (H). (I) Representative endpoint tumor images from C57BL/6 mice bearing AT3‐vector or AT3‐Chst1 tumors treated with clodronate liposomes or control liposomes. (J, K) Tumor growth curves (J) and endpoint tumor weights (K) of AT3‐vector or AT3‐Chst1 tumors in mice treated with clodronate liposomes or control liposomes. Data are presented as mean ± SEM. Statistical significance was determined as described in the Methods. ns, not significant; ^*^ *p* < 0.05; ^**^ *p* < 0.01; ^***^ *p* < 0.001; ^****^ *p* < 0.0001.

To examine the cellular distribution of CHST1 within the breast tumor ecosystem, we analyzed public single‐cell RNA‐seq datasets [17]. CHST1 expression was mainly detected in non‐immune compartments, including malignant epithelial cells and endothelial cells, whereas immune lineages such as macrophages and T cells showed relatively low expression (Figure S4A,B). This pattern suggested that CHST1‐associated immune remodeling was unlikely to be driven by immune cell autonomous CHST1 expression. We therefore examined the relationship between tumor‐associated CHST1 expression and immune infiltration using deconvolution analysis of bulk transcriptomic data. In our cohort, tumors with high CHST1 expression exhibited increased estimated abundance of M2 macrophages (Figure 3A), a pattern that was further supported in the external I‐SPY2 cohort and TIMER 2.0 database (Figure 3B,C).

Given this association, we next tested whether CHST1 expression in tumor cells could directly influence macrophage phenotypes in a non‐contact co‐culture system. PMA‐differentiated THP‐1‐derived macrophages were co‐cultured with genetically manipulated TNBC cells. CHST1 knockdown in MDA‐MB‐468 cells reduced the expression of M2‐like markers in THP‐1‐derived macrophages, whereas CHST1 overexpression in MDA‐MB‐231 cells promoted an M2‐like marker profile (Figure 3D,E and Figure S4C–H). Consistently, CHST1 overexpression enhanced macrophage recruitment (Figure 3F–H). To assess the functional contribution of macrophage‐enriched phagocytic populations in vivo, we used a clodronate liposome–based depletion model. Clodronate liposome treatment markedly attenuated the tumor‐promoting effect of Chst1 overexpression (Figure 3I–K), supporting a functional contribution of phagocytic myeloid populations to Chst1‐associated tumor progression.

Together, these findings suggest that tumor‐cell‐associated CHST1 promotes macrophage recruitment and M2‐like remodeling, thereby contributing to an immunosuppressive tumor microenvironment that supports TNBC progression.

### 2.4 CHST1 Promotes CCL20‐Dependent Macrophage Remodeling Through a Mechanism Largely Uncoupled from Canonical Sulfotransferase Activity

Having established that CHST1 promotes macrophage‐associated immunosuppression, we next sought to determine whether this effect depends on its canonical sulfotransferase activity [18]. Because CHST1 is classically defined as a carbohydrate sulfotransferase, we generated a *Chst1‐R243Q* mutant, a catalytically impaired mutant based on previous reports implicating this residue in sulfotransferase activity, to separate the enzymatic and non‐catalytic functions of CHST1 [19, 20].

**FIGURE 4:**
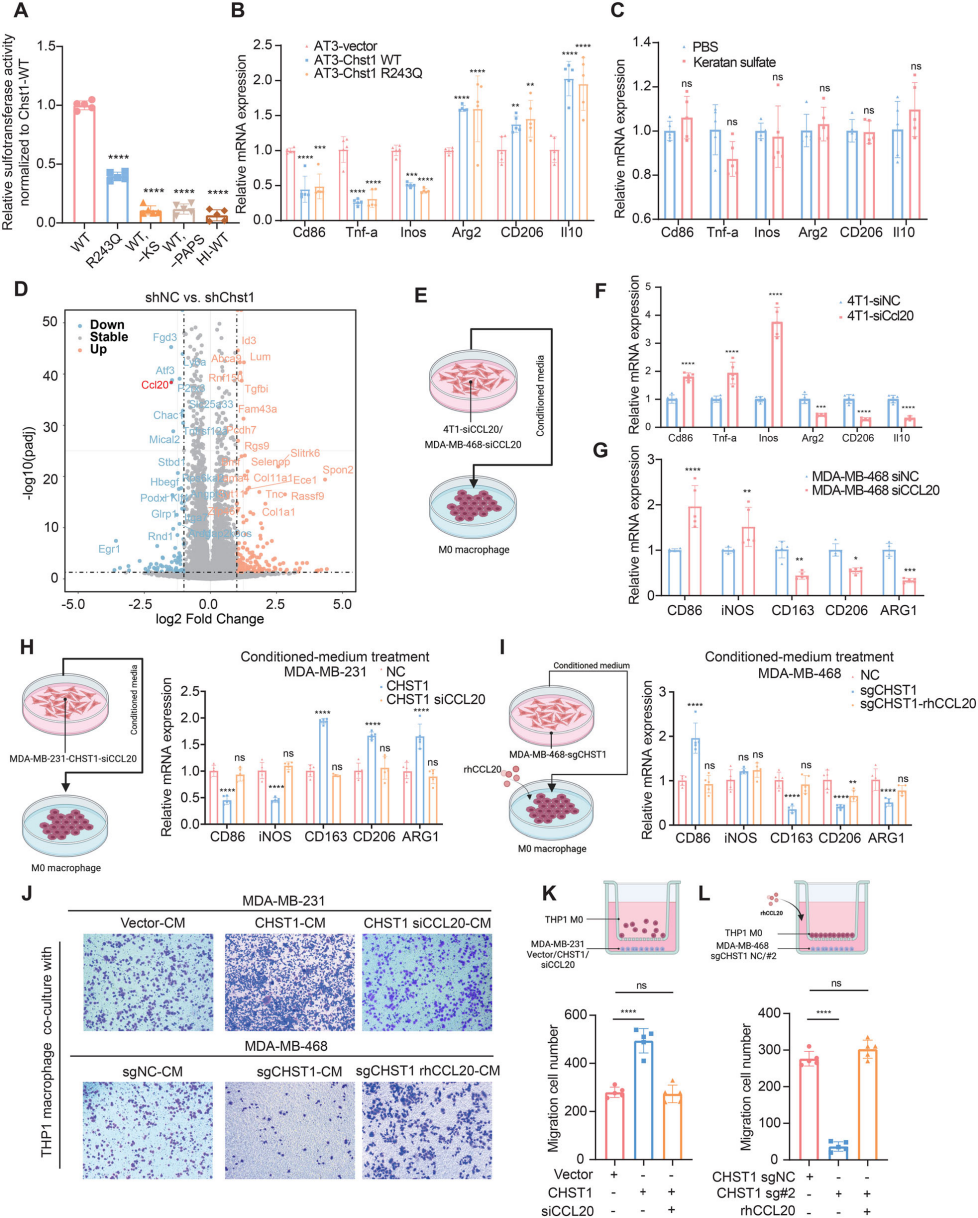
CHST1 promotes CCL20‐dependent macrophage remodeling through a mechanism not primarily dependent on canonical sulfotransferase activity. (A) Relative sulfotransferase activity of immunoprecipitated Chst1‐WT and Chst1‐R243Q. Control reactions included Chst1‐WT without keratan sulfate acceptor substrate (WT −KS), Chst1‐WT without PAPS sulfate donor (WT −PAPS), and heat‐inactivated Chst1‐WT (HI‐WT). Enzymatic activity was normalized to the Chst1‐WT group. (B) RT‐qPCR analysis of macrophage polarization markers in macrophages cultured with conditioned medium from AT3‐vector, AT3‐Chst1‐WT, or AT3‐Chst1‐R243Q cells. M1‐like markers included Cd86, Tnf‐α, and Inos, whereas M2‐like markers included Arg2, Cd206, and Il10. (C) RT‐qPCR analysis of macrophage polarization markers in macrophages treated with PBS or keratan sulfate. (D) Volcano plot showing differentially expressed genes between shNC and shChst1 tumor cells. Ccl20 is highlighted as a downregulated chemokine candidate after Chst1 depletion. (E) Schematic of the conditioned‐medium co‐culture system used to assess the effect of tumor‐cell‐derived CCL20 on macrophage polarization. (F) RT‐qPCR analysis of macrophage polarization markers in macrophages cultured with conditioned medium from 4T1 cells transfected with control siRNA or Ccl20‐targeting siRNA. (G) RT‐qPCR analysis of macrophage polarization markers in PMA‐differentiated THP‐1‐derived macrophages cultured with conditioned medium from MDA‐MB‐468 cells transfected with control siRNA or CCL20‐targeting siRNA. (H) Schematic and RT‐qPCR analysis of macrophage polarization markers in PMA‐differentiated THP‐1‐derived macrophages cultured with conditioned medium from MDA‐MB‐231 cells expressing vector control or CHST1, with or without CCL20 knockdown. (I) Schematic and RT‐qPCR analysis of macrophage polarization markers in PMA‐differentiated THP‐1‐derived macrophages cultured with conditioned medium from MDA‐MB‐468 sgNC or sgCHST1 cells, with or without recombinant human CCL20 supplementation. (J) Representative images of migrated THP‐1‐derived macrophages in Transwell recruitment assays using conditioned medium from MDA‐MB‐231 or MDA‐MB‐468 cells under the indicated conditions. (K) Quantification of migrated THP‐1‐derived macrophages in Transwell recruitment assays using conditioned medium from MDA‐MB‐231 cells expressing vector control or CHST1, with or without CCL20 knockdown. (L) Quantification of migrated THP‐1‐derived macrophages in Transwell recruitment assays using conditioned medium from MDA‐MB‐468 sgNC or sgCHST1 cells, with or without recombinant human CCL20 supplementation. Data are presented as mean ± SEM. Statistical significance was determined as described in the Methods. ns, not significant; ^*^ *p* < 0.05; ^**^ *p* < 0.01; ^***^ *p* < 0.001; ^****^ *p* < 0.0001.

We first verified the enzymatic consequence of this mutation in the TNBC experimental system. Flag‐tagged Chst1‐WT and *Chst1‐R243Q* were expressed under matched conditions and enriched by anti‐Flag immunoprecipitation, with comparable protein recovery confirmed before the enzymatic assay (Figure S5A). Because CHST1 is classically characterized as a keratan sulfate Gal‐6‐sulfotransferase, the immunoprecipitated Chst1 proteins were subjected to 3′‐phosphoadenosine‐5′‐phosphate (PAP)‐based sulfotransferase activity assay using 3′‐phosphoadenosine‐5′‐phosphosulfate (PAPS) as the sulfate donor and keratan sulfate as the acceptor substrate [20]. Chst1‐WT showed robust sulfotransferase activity, whereas *Chst1‐R243Q* exhibited markedly reduced activity. In contrast, reactions lacking keratan sulfate, lacking PAPS, or using heat‐inactivated Chst1‐WT produced only low background‐level signals, confirming that the assay reflected a complete Chst1‐mediated sulfotransferase reaction (Figure 4A). These data demonstrate that *R243Q* substantially impairs Chst1 catalytic activity in our TNBC model.

We next asked whether the marked reduction in catalytic activity of *Chst1‐R243Q* was sufficient to abolish the macrophage‐regulatory function of CHST1. Conditioned medium from Chst1‐WT‐expressing tumor cells shifted macrophage marker expression toward an M2‐like pattern, characterized by reduced M1‐like markers and increased M2‐like markers. Notably, conditioned medium from *Chst1‐R243Q*‐expressing cells largely retained this macrophage‐regulatory activity despite markedly impaired sulfotransferase activity (Figure 4B). In contrast, exogenous keratan sulfate supplementation did not significantly alter macrophage marker expression (Figure 4C). These findings suggest that CHST1‐associated macrophage remodeling is not primarily explained by canonical CHST1 sulfotransferase activity.

To identify downstream mediators of this non‐canonical macrophage‐regulatory effect, we performed differential expression analysis with a focus on secreted immune‐regulatory factors, given their established roles in macrophage recruitment and polarization [21, 22, 23]. This analysis revealed that Chst1 knockdown markedly downregulated Ccl20, a chemokine implicated in macrophage polarization and immune‐cell trafficking (Figure 4D) [24]. We therefore focused on CCL20 as a potential downstream mediator linking CHST1 to macrophage remodeling.

We first examined whether CCL20 depletion alone was sufficient to alter the macrophage‐regulatory activity of tumor‐cell conditioned medium. Ccl20 knockdown in 4T1 cells shifted macrophage marker expression away from an M2‐like profile, with increased M1‐like markers and reduced M2‐like markers (Figure 4E,F). A similar pattern was observed after CCL20 knockdown in MDA‐MB‐468 cells, where conditioned medium showed a reduced capacity to maintain M2‐like macrophage marker expression (Figure 4G). These results indicate that tumor‐cell‐derived CCL20, contributes to the macrophage‐polarizing activity of conditioned medium.

We then performed complementary perturbation and rescue experiments to place CCL20 downstream of CHST1. In the CHST1 gain‐of‐function setting, CHST1 overexpression enhanced the ability of tumor‐cell conditioned medium to induce M2‐like macrophage features, whereas siRNA‐mediated CCL20 knockdown attenuated this effect (Figure 4H and Figure S5B). Conversely, in the CHST1 loss‐of‐function setting, CHST1 deficiency weakened the macrophage‐regulatory activity of conditioned medium, and recombinant human CCL20 partially restored the M2‐like marker profile (Figure 4I). Together, these results support CCL20 as an important downstream mediator of CHST1‐associated tumor–macrophage crosstalk.

Consistent with these marker‐expression changes, Transwell assays showed that conditioned medium from CHST1‐overexpressing cells enhanced THP‐1‐derived macrophage migration, whereas CCL20 knockdown reduced this effect (Figure 4J,K). Conversely, conditioned medium from CHST1‐deficient cells showed reduced macrophage‐recruiting activity, which was restored by recombinant CCL20 supplementation (Figure 4J,L). Collectively, these findings support a non‐canonical CHST1–CCL20 axis that promotes macrophage recruitment and M2‐like remodeling through a mechanism largely uncoupled from canonical CHST1 sulfotransferase activity.

### 2.5 CHST1 Associates With NKRF and Is Linked to Altered NKRF Localization and CCL20 Expression

**FIGURE 5:**
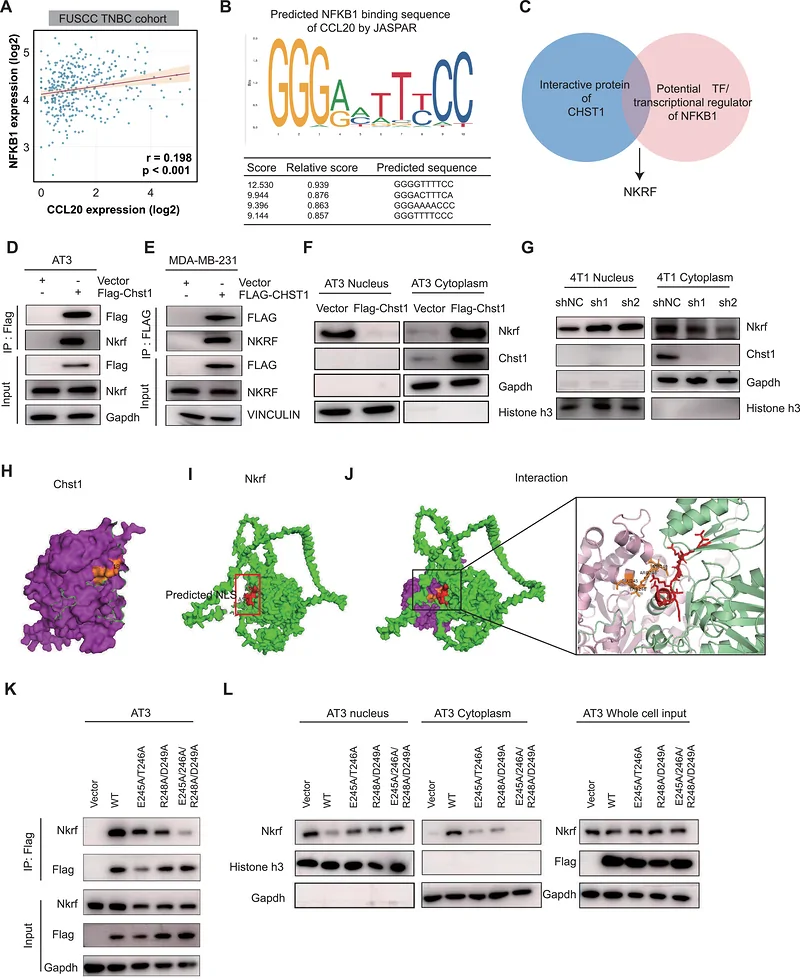
CHST1 associates with NKRF and supports a localization‐dependent model of CCL20 regulation. (A) Correlation between NFKB1 and CCL20 mRNA expression in the FUSCC TNBC cohort. (B) JASPAR‐based prediction of putative NF‐κB/NFKB1‐binding motifs within the CCL20 promoter region. (C) Schematic overview of the strategy used to identify CHST1‐associated candidate regulators potentially linked to NF‐κB‐responsive transcription. (D, E) Co‐IP analysis validating the association between Flag‐Chst1/CHST1 and endogenous Nkrf/NKRF in representative murine and human TNBC gain‐of‐function models, including AT3 cells (D) and MDA‐MB‐231 cells (E). (F, G) Nuclear‐cytoplasmic fractionation analysis of Nkrf distribution in AT3‐Chst1 overexpression cells(F) and 4T1‐shChst1 cells(G). (H) AlphaFold3‐predicted structural model of Chst1, with candidate interface residues highlighted. (I) AlphaFold3‐predicted structural model of Nkrf, with the predicted nuclear localization signal (NLS) region highlighted. (J) AlphaFold3‐predicted Chst1–Nkrf complex showing the spatial proximity between the predicted Chst1 interface and the predicted Nkrf NLS region. (K) Co‐IP analysis of endogenous Nkrf association with wild‐type Chst1 or the indicated Chst1 interface mutants in AT3 cells. (L) Nuclear–cytoplasmic fractionation analysis of Nkrf distribution in AT3 cells expressing vector control, wild‐type Chst1, or the indicated Chst1 interface mutants. Histone H3 and GAPDH were used as nuclear and cytoplasmic markers, respectively. Whole‐cell lysates were analyzed as input controls.

To define how CHST1 regulates CCL20 expression, we first considered whether CHST1 could directly control CCL20 transcription. Given that CHST1 is classically characterized as a carbohydrate sulfotransferase and lacks recognizable DNA‐binding or transcription factor‐like domains, we reasoned that CHST1 was unlikely to directly regulate CCL20 transcription through a canonical DNA‐binding mechanism [20]. We therefore hypothesized that CHST1 may influence CCL20 transcription indirectly through an intermediate transcriptional regulatory pathway. Because CCL20 has been reported as an NF‐κB‐responsive chemokine [25, 26], we used this established regulatory relationship as a mechanistic framework to explore how CHST1 might influence CCL20 transcription. In the FUSCC TNBC cohort, NFKB1 expression was positively correlated with CCL20 expression (Figure 5A), and JASPAR‐based promoter motif analysis identified putative NF‐κB‐binding motifs within the CCL20 promoter region (Figure 5B). Together, these observations supported the relevance of an NF‐κB‐associated CCL20 transcriptional program in TNBC and prompted us to search for CHST1‐associated regulators capable of modulating NF‐κB‐responsive transcription.

To identify such regulators, we performed immunoprecipitation coupled with mass spectrometry (IP‐MS) to profile CHST1‐associated proteins. Among the candidate interactors, NF‐κB‐repressing factor (NKRF) was of particular interest because it has been reported to repress NF‐κB‐responsive genes by binding to negative regulatory elements (NREs) and limiting NF‐κB‐mediated transcriptional activation (Figure 5C) [27, 28]. Consistent with the IP‐MS results, Co‐IP assays validated the association between CHST1/Chst1 and NKRF in representative murine and human TNBC gain‐of‐function models, AT3 and MDA‐MB‐231, respectively (Figure 5D,E).

Because the transcriptional repressive function of NKRF depends on its access to the nuclear compartment, we next asked whether Chst1 regulates NKRF abundance or its subcellular distribution. In complementary Chst1 loss‐ and gain‐of‐function models, reverse‐transcription quantitative PCR (RT‐qPCR) analysis showed that neither Chst1 depletion nor Chst1 overexpression significantly altered Nkrf mRNA expression. Consistently, Western blotting of whole‐cell lysates showed largely comparable total NKRF protein levels despite efficient Chst1 knockdown or overexpression (Figure S6A–D). These findings indicate that Chst1 does not primarily regulate NKRF at the transcriptional or total protein‐expression level.

We then examined whether Chst1 affects NKRF subcellular localization. Nuclear–cytoplasmic fractionation showed that Chst1 overexpression increased cytoplasmic retention of NKRF and reduced its nuclear accumulation, whereas Chst1 depletion promoted NKRF nuclear accumulation (Figure 5F,G). These results support a localization‐dependent mechanism by which Chst1 modulates NKRF function without substantially altering total NKRF abundance.

To further explore the structural basis of this localization‐dependent regulation, we performed AlphaFold3‐based modeling of the CHST1–NKRF complex. The predicted CHST1–NKRF interface was spatially proximal to the predicted nuclear localization signal (NLS) region of NKRF (Figure 5H–J). We therefore generated CHST1 interface mutants targeting residues within the predicted interface, including E245A/T246A, R248A/D249A, and the combined E245A/T246A/R248A/D249A mutant. Co‐IP assays showed that wild‐type CHST1 associated with NKRF, whereas mutation of the predicted interface residues weakened this association, with the combined mutant showing the most pronounced reduction (Figure 5K). Functionally, these interface mutations attenuated CHST1‐mediated NKRF cytoplasmic retention and partially restored nuclear NKRF accumulation compared with wild‐type CHST1 (Figure 5L).

Together, these findings support a model in which CHST1 associates with NKRF through a predicted NLS‐proximal interface and restricts NKRF nuclear accumulation. This localization change may reduce NKRF‐mediated repression and contribute to increased activity of an NF‐κB‐associated CCL20 transcriptional program (Figure S6E).

### 2.6 Pharmacologic CCR6 Blockade Enhances TNBC Responsiveness to Anti–PD‐1 Therapy

Having identified CCL20 as an important downstream mediator of CHST1‐associated macrophage remodeling, we next asked whether therapeutic blockade of this chemokine axis could improve TNBC responsiveness to immune checkpoint inhibition. Because CCL20 signals through its cognate receptor CCR6 [29], we evaluated a CCR6 antagonist in combination with anti–PD‐1 therapy in syngeneic TNBC models.

**FIGURE 6:**
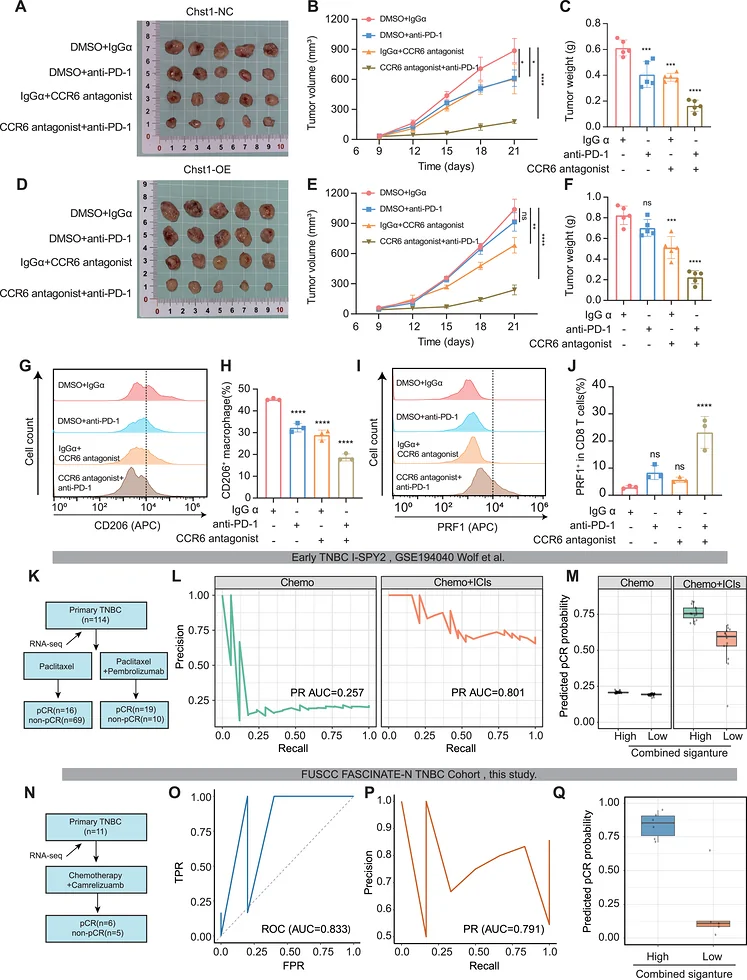
Targeting the CHST1–CCL20–CCR6 axis enhances TNBC responsiveness to anti–PD‐1 therapy and provides a candidate response‐prediction signature. (A–C) 66cl4 mouse breast cancer cells stably expressing vector control were implanted into BALB/c mice, followed by treatment with Vehicle (DMSO) plus IgG2a isotype control, Vehicle (DMSO) plus anti–PD‐1, CCR6 antagonist plus IgG2a isotype control, or CCR6 antagonist plus anti–PD‐1. Representative endpoint tumors are shown in A. Tumor growth curves (B) and endpoint tumor weights (C) were measured. (D–F) 66cl4 mouse breast cancer cells stably overexpressing Chst1 were implanted into BALB/c mice and treated as described above. Representative endpoint tumors are shown in D. Tumor growth curves (E) and endpoint tumor weights (F) were measured. (G, H) Representative flow cytometry histograms of CD206 expression in tumor‐infiltrating macrophages (G) and quantification of CD206^+^ macrophages among tumor‐infiltrating macrophages (H) under the indicated treatment conditions. (I, J) Representative flow cytometry histograms of PRF1 expression in tumor‐infiltrating CD8^+^ T cells (I) and quantification of PRF1^+^ CD8^+^ T cells (J) under the indicated treatment conditions. (K) Overview of the I‐SPY2 early TNBC cohort, including patients treated with paclitaxel alone or paclitaxel plus pembrolizumab. (L, M) Predictive performance of the CHST1–NKRF–CCL20 combined signature in the I‐SPY2 cohort, assessed by precision–recall curves (L) and predicted pCR probability in high‐ and low‐signature groups across different treatment arms (M). (N) Overview of the FASCINATE‐N TNBC cohort from this study. (O–Q) Predictive performance of the CHST1–NKRF–CCL20 combined signature in the FASCINATE‐N cohort, assessed by receiver operating characteristic curve analysis (O), precision–recall curve analysis (P), and predicted pCR probability in high‐ and low‐signature groups (Q). Data are presented as mean ± SEM unless otherwise indicated. Statistical significance was determined as described in the Methods. ns, not significant; ^*^ *p* < 0.05; ^**^ *p* < 0.01; ^***^ *p* < 0.001; ^****^ *p* < 0.0001.

We first tested this strategy in an independent 66cl4 model using control and Chst1‐overexpressing tumor cells. Chst1 overexpression was confirmed before in vivo implantation (Figure S7A). Tumor‐bearing mice were treated with vehicle plus IgG control, anti–PD‐1 monotherapy, CCR6 antagonist monotherapy, or combined CCR6 antagonist and anti–PD‐1 therapy. In control tumors, anti–PD‐1 or CCR6 antagonist monotherapy produced partial tumor suppression, whereas the combination treatment resulted in more pronounced inhibition of tumor growth and endpoint tumor weight (Figure 6A–C). In Chst1‐overexpressing tumors, anti–PD‐1 monotherapy showed limited antitumor efficacy, consistent with reduced responsiveness of Chst1‐high tumors to PD‐1 blockade. By contrast, CCR6 antagonist treatment attenuated tumor progression, and combined CCR6 blockade with anti–PD‐1 therapy further suppressed both tumor growth and endpoint tumor burden (Figure 6D–F). These findings suggest that Chst1 overexpression is associated with impaired responsiveness to anti–PD‐1 therapy, whereas blockade of the CCL20–CCR6 axis can improve therapeutic sensitivity, particularly in Chst1‐high tumors. An additional AT3‐based syngeneic model further supported the benefit of combining CCR6 blockade with anti–PD‐1 therapy (Figure S7B–G). Consistent with the proposed immune‐remodeling mechanism, immunophenotyping of treated tumors showed that CCR6 blockade, especially in combination with anti–PD‐1, reduced CD206^+^ macrophage accumulation and enhanced cytotoxic CD8^+^ T‐cell activity, as reflected by increased Perforin and Granzyme B expression (Figure 6G–J and Figure S7H–I). Together, these in vivo data provide proof‐of‐concept evidence that targeting the CHST1–CCL20–CCR6 axis can alleviate CHST1‐associated immunosuppression and enhance TNBC responsiveness to immune checkpoint inhibition.

To further explore the translational relevance of this pathway, we developed a composite expression signature based on CHST1, NKRF, and CCL20, aiming to capture the activity of the CHST1–NKRF–CCL20 regulatory axis in clinical TNBC samples. We first evaluated this signature in an external early‐stage TNBC cohort with available treatment‐response data. Notably, the signature showed limited discrimination for chemotherapy response but improved predictive performance for response to chemoimmunotherapy, suggesting that it may be more closely associated with immune‐dependent therapeutic sensitivity than with general chemosensitivity (Figure 6K–M). We then assessed the signature in our internal FASCINATE‐N cohort of TNBC patients treated with neoadjuvant chemoimmunotherapy. Consistently, the CHST1–NKRF–CCL20 expression signature showed favorable discrimination of pathological complete response, supporting its potential value for stratifying patients according to immunotherapy responsiveness (Figure 6N–Q). Together, these findings suggest that the CHST1–NKRF–CCL20 axis is not only mechanistically linked to immune resistance but may also provide a candidate transcriptomic biomarker for identifying TNBC patients more likely to benefit from immunotherapy‐based treatment. The raw immunoblot images corresponding to all immunoblots presented in this study are provided in Figure S8.

## 3 Discussion

In this study, by leveraging baseline transcriptomic data from a TNBC neoadjuvant immunotherapy cohort, we identified CHST1 as a candidate driver of therapeutic resistance. Mechanistically, our findings support a non‐canonical regulatory model involving CHST1, NKRF, and CCL20. CHST1 associates with NKRF and is linked to its cytoplasmic retention and reduced nuclear accumulation. These localization changes may weaken NKRF‐mediated repression and contribute to increased NF‐κB‐associated CCL20 expression. CCL20 perturbation and rescue experiments support a functional contribution of tumor‐cell‐derived CCL20 to macrophage remodeling. From a translational perspective, pharmacologic blockade of CCR6 enhanced the antitumor activity of anti–PD‐1 therapy in syngeneic models, and a CHST1–NKRF–CCL20 expression signature showed potential value for stratifying immunotherapy response.

Conventionally, CHST1 is recognized as a Golgi‐resident carbohydrate sulfotransferase that catalyzes keratan sulfate synthesis and shapes sulfated glycan epitopes involved in extracellular matrix interactions and cell–microenvironment communication [20, 30]. Although elevated CHST1 expression has been associated with poor outcomes in several malignancies, the molecular basis for its immunomodulatory function has remained poorly defined [31, 32]. Our findings extend the classical view of CHST1 by revealing a non‐canonical immune‐regulatory function that is largely uncoupled from its canonical sulfotransferase activity. In support of this interpretation, the catalytically impaired *Chst1‐R243Q* mutant retained macrophage‐regulatory activity despite markedly reduced sulfotransferase activity, suggesting that CHST1 can regulate immune remodeling through a scaffold‐like or localization‐dependent mechanism rather than solely through glycan sulfation. This observation is consistent with the broader concept of “moonlighting” functions in cancer biology, in which proteins traditionally assigned to metabolic or enzymatic pathways acquire additional non‐catalytic roles in signaling or transcriptional regulation [33, 34].

Importantly, the enzyme‐activity‐independent component described here does not exclude a contribution from canonical CHST1‐mediated glycan sulfation. Instead, these two layers of CHST1 function may operate through distinct but potentially interconnected mechanisms. The CHST1–NKRF association is accompanied by reduced nuclear NKRF accumulation, which may contribute to diminished NKRF‐mediated repression and increased CCL20 expression. In parallel, CHST1‐dependent glycan sulfation may still influence tumor–microenvironment interactions by modulating extracellular matrix organization, chemokine presentation, receptor–ligand accessibility, or immune‐cell trafficking. Thus, the CHST1–NKRF mechanism may function independently of, in parallel with, or potentially in cooperation with canonical CHST1‐mediated glycan sulfation to shape the immunosuppressive microenvironment. Future studies integrating catalytic mutants, localization mutants, compartment‐specific CHST1 perturbation, and glycomic profiling will be needed to determine how these two functional layers interact and whether one predominates in specific cellular or microenvironmental contexts. In TNBC, CHST1 may therefore be co‐opted to facilitate immune microenvironmental reprogramming, contrasting with the primarily proliferative roles reported in other tumor types [31].

Tumor–microenvironment crosstalk is a major determinant of immunotherapy response. Among the immune populations within the tumor microenvironment, tumor‐associated macrophages, particularly M2‐like macrophages, are important mediators of immune checkpoint blockade resistance. These cells can suppress antitumor immunity by producing immunosuppressive cytokines such as IL‐10 and TGF‐β, limiting cytotoxic CD8^+^ T‐cell infiltration or function, and contributing to immune‐excluded or immune‐suppressed tumor states. Previous studies have implicated several tumor‐derived factors, including CSF‐1 and CCL5, in macrophage recruitment and polarization [35, 36]. Our work highlights the CCL20–CCR6 axis as an additional chemokine‐mediated mechanism of macrophage‐associated immune suppression in CHST1‐high TNBC. More broadly, macrophage‐centered immune remodeling can be shaped by either immunosuppressive or immunostimulatory signaling programs. For example, recent work showed that combined targeting of STING, TGF‐β, and PD‐L1 enhances antitumor immunity through a CXCL16–CXCR6 chemokine program involving macrophages and dendritic cells, thereby supporting cytotoxic T‐cell recruitment and maintenance [37]. In this context, the CHST1–CCL20–CCR6 axis described here may represent a complementary but functionally opposite tumor‐cell‐intrinsic chemokine program, one that favors M2‐like macrophage accumulation and immunosuppressive remodeling rather than cytotoxic T‐cell‐supportive myeloid activation. Future studies should determine whether immunostimulatory approaches such as STING activation can counteract CHST1‐driven CCL20–CCR6‐mediated macrophage remodeling, and whether CHST1‐high tumors show distinct sensitivity to STING‐based immunotherapy combinations.

Although our experimental analyses focused primarily on macrophages and cytotoxic CD8^+^ T‐cell features, CHST1‐driven CCL20–CCR6 signaling may contribute to broader immunosuppressive remodeling of the tumor microenvironment. Beyond macrophages, CCL20–CCR6 signaling can potentially affect additional CCR6‐responsive immune subsets in a context‐dependent manner. Moreover, M2‐like macrophages may secondarily reinforce immune suppression by producing immunoregulatory cytokines, limiting cytotoxic T‐cell activity, and supporting regulatory immune programs. Therefore, suppressive populations such as Tregs, MDSCs, or other myeloid subsets may also be influenced downstream of CHST1‐associated macrophage remodeling. Nevertheless, the current study was designed to interrogate the macrophage/CD8^+^ T‐cell axis and did not include dedicated marker panels for these additional immune populations. Thus, our data do not directly define whether Tregs, MDSCs, or other suppressive subsets participate in CHST1‐driven immune remodeling. Future studies using expanded multicolor flow cytometry, single‐cell transcriptomics, and spatial immune profiling will be needed to determine whether the CHST1–CCL20–CCR6 axis coordinates a broader suppressive immune network beyond macrophages.

Mechanistically, our findings support a localization‐dependent regulatory model involving CHST1, NKRF, and an NF‐κB‐associated CCL20 transcriptional program. CHST1 associates with NKRF and promotes its cytoplasmic retention without substantially altering total NKRF abundance. This localization change may reduce the nuclear availability of NKRF and thereby contribute to increased CCL20 expression. However, because NKRF or NF‐κB occupancy at the CCL20 promoter and CCL20 promoter activity were not directly assessed, the proposed transcriptional sequence remains inferential. Similarly, although tumor‐cell CCL20 perturbation and rescue experiments support a functional role for CCL20 in macrophage remodeling, and systemic CCR6 blockade improved therapeutic response in vivo, the current data do not establish macrophage‐intrinsic CCR6 as the direct receptor dependency underlying these effects. Future studies incorporating NKRF and NF‐κB ChIP‐qPCR, CCL20 promoter‐reporter assays, NKRF rescue experiments, and macrophage‐specific CCR6 perturbation will be required to resolve these mechanistic links.

AlphaFold3‐guided interface‐mutant experiments further supported the functional relevance of this model. Mutations of CHST1 residues within the predicted NLS‐proximal CHST1–NKRF interface weakened the CHST1–NKRF association and attenuated CHST1‐mediated NKRF cytoplasmic retention, leading to partial restoration of nuclear NKRF accumulation. These findings suggest that CHST1 may restrict NKRF nuclear entry, at least in part, through an interface located near the predicted NKRF NLS region. Nevertheless, this model should not be interpreted as evidence that CHST1 globally activates NF‐κB signaling. Rather, our data are consistent with a context‐dependent model in which reduced nuclear NKRF availability may contribute to CCL20 upregulation. Future studies incorporating promoter occupancy assays, higher‐resolution structural analyses, and broader profiling of NF‐κB‐responsive transcriptional outputs will be needed to further define how the CHST1–NKRF interaction selectively shapes downstream inflammatory programs.

From a therapeutic perspective, our findings suggest that the CCL20–CCR6 axis may represent a potential intervention point for mitigating CHST1‐associated immune suppression. In syngeneic murine models, pharmacologic CCR6 inhibition enhanced the antitumor activity of PD‐1 blockade and was accompanied by reduced M2‐like macrophage features and increased cytotoxic CD8^+^ T‐cell activity. These results provide proof‐of‐concept evidence that blockade of the downstream CCL20–CCR6 axis can partially counteract CHST1‐driven immunosuppressive remodeling. Nevertheless, several translational issues remain. The CCR6 antagonist regimen used in this study was selected as a literature‐informed proof‐of‐concept schedule and was not formally optimized in TNBC models. Future studies will be required to define the optimal dose, dosing interval, pharmacodynamic inhibition, and safety window of CCR6‐axis blockade, particularly when CCR6 blockade is combined with PD‐1 inhibition, because broader changes in immune‐cell recruitment and activation could influence both antitumor immunity and immune‐related toxicity. Direct targeting of CHST1 may also be challenging because of its Golgi localization, the need for intracellular accessibility, selectivity among sulfotransferase family members, and potential effects on physiological glycan sulfation. Thus, although the CHST1–CCL20–CCR6 axis represents a biologically supported therapeutic concept, additional preclinical optimization and safety evaluation will be needed before clinical translation. In this context, the CHST1–NKRF–CCL20 expression signature may provide a complementary biomarker strategy for patient stratification and may help identify patients who could benefit from rational combination approaches, pending prospective validation.

Several limitations should be acknowledged. First, the discovery analysis was performed in a relatively small cohort, and although the findings were supported by multiple external datasets, prospective validation in larger and well‐annotated clinical cohorts will be required. Second, the CCR6‐inhibition combination strategy has thus far been evaluated only in murine models; its translational feasibility, safety profile, and clinical efficacy remain to be established. Third, although our data support a localization‐dependent CHST1–NKRF mechanism, the structural basis by which CHST1 restricts NKRF nuclear trafficking remains incompletely resolved and will require higher‐resolution structural and functional interrogation.

## 4 Conclusions

In conclusion, this study identifies CHST1 as a candidate mediator of immunotherapy resistance in TNBC and reveals an immune‐context‐dependent role for CHST1 in shaping an immunosuppressive tumor microenvironment. Our findings support a non‐canonical regulatory model in which CHST1 associates with NKRF, limits its nuclear accumulation, and contributes to increased expression of the NF‐κB‐responsive chemokine CCL20. Tumor‐cell‐derived CCL20, in turn, promotes macrophage recruitment and M2‐like remodeling, thereby impairing cytotoxic T‐cell activity. Notably, this macrophage‐regulatory function was largely retained by a catalytically impaired CHST1 mutant, suggesting that it is not primarily dependent on canonical sulfotransferase activity. Pharmacologic blockade of the CCL20–CCR6 axis enhanced the antitumor efficacy of anti–PD‐1 therapy in syngeneic TNBC models. Together, these findings establish the CHST1–NKRF–CCL20–macrophage axis as a potential mechanism of immune resistance and provide a rationale for evaluating CCR6‐targeted combinations and the CHST1–NKRF–CCL20 signature in future translational studies.

## 5 Methods

### 5.1 Patients and Clinical Cohorts

This study was conducted in accordance with the Declaration of Helsinki and approved by the Institutional Review Board (IRB) of Fudan University Shanghai Cancer Center (FUSCC; Approval No. 2209261–7). Written informed consent was obtained from all participants prior to enrollment. The discovery cohort comprised 11 patients with triple‐negative breast cancer from the prospective FASCINATE‐N trial (ClinicalTrials.gov identifier: NCT05582499). All patients in this cohort received neoadjuvant chemo‐immunotherapy, and pre‐treatment tumor tissue samples were collected for transcriptomic profiling. Detailed clinicopathological characteristics are provided in Table S1. For independent validation, we utilized publicly available datasets, including the I‐SPY2 TNBC immunotherapy cohort (GSE194040), The Cancer Genome Atlas (TCGA) TNBC dataset, and the retrospective FUSCC TNBC cohort (GSE118527).

### 5.2 Bioinformatics Analysis

Bulk RNA‐seq data from the discovery cohort were processed using standard bioinformatics pipelines. Differential expression analysis between the pathological complete response and non‐pCR groups was performed using the DESeq2 package (v1.42.1) in R. Differentially expressed genes (DEGs) were identified based on a significance threshold of |log2(fold change)| > 2 and an adjusted *p*‐value < 0.05. To identify robust predictors of immunotherapy resistance, the Least Absolute Shrinkage and Selection Operator (LASSO) regression algorithm was implemented using the glmnet package (v4.1‐10). Functional enrichment analysis was performed using the clusterProfiler package in R with gene sets obtained from the Gene Ontology database. The relative abundance of immune cell populations within the tumor microenvironment was deconvoluted using the CIBERSORTx algorithm. For external validation, predictive performance was evaluated using Receiver Operating Characteristic (ROC) curve analysis. Survival outcomes were assessed via Kaplan–Meier analysis with the log‐rank test. Correlations between gene signatures and immune features were determined using Pearson or Spearman correlation coefficients.

### 5.3 Cell Lines and Culture

The human TNBC cell lines (MDA‐MB‐231, MDA‐MB‐468) and the murine TNBC cell line 4T1 were obtained from the American Type Culture Collection (ATCC; Manassas, VA, USA). The murine TNBC cell line AT3, 66cl4 were kindly provided by Fudan University Shanghai Cancer Center. The murine macrophage cell line RAW264.7 and the human monocytic cell line THP‐1 were purchased from ATCC. Cell lines were authenticated using short tandem repeat (STR) profiling and were routinely tested negative for mycoplasma contamination. Cells were cultured in DMEM/RPMI‐1640 supplemented with 10% fetal bovine serum (FBS) and 1% penicillin‐streptomycin in a humidified incubator at 37°C with 5% CO_2_. All cell lines were cultured for a limited number of passages after thawing and were routinely tested to confirm the absence of mycoplasma contamination. Unless otherwise stated, cells used for in vitro functional assays, including proliferation, migration/invasion, co‐culture, chemotaxis, RT‐qPCR, Western blotting, and Co‐IP experiments, were maintained between passages P3–P20 after thawing. Cells used for in vivo implantation experiments were maintained between passages P3–P15 after thawing and were harvested during the logarithmic growth phase to ensure viability and experimental consistency. Cell lines were not used beyond the indicated passage range.

### 5.4 Transfection and Viral Infection

Constructs targeting CHST1 were designed with distinct strategies based on species: small guide RNAs (sgRNAs) were utilized for CRISPR/Cas9‐mediated knockout in MDA‐MB‐468 cell lines, while short hairpin RNAs (shRNAs) were employed for knockdown in 4T1 cell lines. Plasmids encoding CHST1/Chst1 overexpression, the catalytically impaired Chst1‐R243Q mutant, the predicted interface mutants Chst1‐E245A/T246A, Chst1‐R248A/D249A, and Chst1‐E245A/T246A/R248A/D249A, human CHST1‐targeting sgRNAs, and murine Chst1‐targeting shRNAs were purchased from YiXueSheng Biosciences Inc. (Shanghai, China). Additionally, small interfering RNAs (siRNA) targeting CCL20 were obtained from the same vendor. For the generation of stable cell lines, HEK‐293T cells were co‐transfected with the respective lentiviral vectors and packaging plasmids (psPAX2 and pMD2.G) using a transfection reagent (Vazyme, #TL201‐01/02). Viral supernatants were collected 48 h post‐transfection, filtered through a 0.45‐µm syringe filter, and applied to target TNBC cells for 24 h. Stable transfectants were selected using puromycin for at least 3 days. For transient silencing of CCL20, cells were transfected with specific siRNAs using the transfection reagent. The efficiency of knockout, knockdown, and overexpression was verified by Western blot and RT‐qPCR.

### 5.5 Real‐Time Quantitative PCR and Western Blot

Total RNA was extracted using TRIzol reagent (Invitrogen, #15596018), reverse‐transcribed to cDNA using the HiScript III RT SuperMix (Vazyme, #R333‐01), and quantified using SYBR Green Master Mix (Vazyme, #Q711) on the QuantStudio system. Gene expression was normalized to GAPDH. For Western blotting, proteins were extracted, separated by SDS‐PAGE, transferred onto PVDF membranes, and incubated with the indicated primary antibodies, followed by HRP‐conjugated secondary antibodies. Protein signals were detected by chemiluminescence. Primer sequences are listed in Supplementary Table S2, and detailed antibody information is provided in Supplementary Table S3.

### 5.6 Sulfotransferase Activity Assay

To assess Chst1 sulfotransferase activity, Flag‐tagged wild‐type Chst1 or *Chst1‐R243Q* was expressed in TNBC cells under matched experimental conditions. Chst1 expression was confirmed by Western blotting, and Flag‐tagged Chst1 proteins were enriched by anti‐Flag immunoprecipitation. The immunoprecipitated Chst1‐containing samples were then subjected to an in vitro sulfotransferase reaction using keratan sulfate as the acceptor substrate and PAPS as the sulfate donor, based on the canonical activity of CHST1 as a keratan sulfate Gal‐6‐sulfotransferase [20]. Sulfotransferase activity was quantified using a PAP‐based phosphatase‐coupled colorimetric assay with the Universal Sulfotransferase Activity Kit (R&D Systems/Bio‐Techne, Cat. No. EA003) according to the manufacturer's instructions. Control reactions were performed using wild‐type Chst1 with omission of keratan sulfate, omission of PAPS, or heat inactivation before the enzymatic reaction. Absorbance values were background‐subtracted and normalized to the wild‐type Chst1 group.

### 5.7 In Vitro Functional Assays

Cell proliferation was assessed using the Cell Counting Kit‐8 (CCK‐8; Beyotime, #C0039) and colony formation assays according to the manufacturer's protocols. For tumor cell migration and invasion assays, cells were seeded into 8‐µm pore‐size Transwell chambers (Corning, NY, USA), with the upper chambers pre‐coated with Matrigel (Corning, #354248) for invasion assays.

### 5.8 Macrophage Polarization and Recruitment Assays

THP‐1 cells were differentiated into macrophage‐like cells by PMA treatment. For macrophage polarization assays, THP‐1‐derived macrophages or RAW264.7 cells were cultured with genetically manipulated TNBC cells in a non‐contact Transwell system or treated with tumor‐cell‐conditioned medium, as indicated. Where specified, CCL20 knockdown, recombinant CCL20 supplementation, or keratan sulfate treatment was performed. After 48 h, macrophages were collected, and the expression of M1‐ and M2‐like macrophage markers was assessed by RT‐qPCR. Macrophage recruitment was evaluated using Transwell inserts with an 8‐µm pore size. Macrophages were seeded in the upper chamber, while tumor cells or tumor‐cell‐conditioned medium under the indicated experimental conditions were placed in the lower chamber. After incubation, migrated cells were fixed, stained with crystal violet, imaged, and quantified in randomly selected microscopic fields.

### 5.9 In Vivo Tumor Models and Treatment Regimens

All animal experiments were approved by the Institutional Animal Care and Use Committee (IACUC) of Fudan University and conducted in strict accordance with institutional guidelines. Female BALB/c mice, C57BL/6 mice, and B‐NDG nude mice (6–8 weeks old) were utilized. For syngeneic tumor models, 4T1 and 66cl4 cells were injected into the mammary fat pads of BALB/c mice, while AT3 TNBC cells were injected into C57BL/6 mice.

### 5.10 Macrophage Depletion and Combination Therapy

For macrophage depletion studies, mice received intraperitoneal (i.p.) injections of 200 µL Clodronate Liposomes (Yeasen, Shanghai, China; #40337ES010) or control liposomes on days 1 and 7 post‐inoculation, followed by maintenance doses of 100 µL every 5 days. For immunotherapy combination experiments, syngeneic 66cl4 and AT3 models were used. The 66cl4 model was used as the validation model, whereas the AT3 model provided additional supportive evidence. 66cl4 control or Chst1‐overexpressing cells were implanted into BALB/c mice, and AT3 control or Chst1‐overexpressing cells were implanted into C57BL/6 mice. Mice were randomized into 8 groups (n = 5 per group) based on tumor genotype and treatment:Vehicle + Isotype: DMSO (s.c.) + IgG2a isotype control (i.p.); Vehicle + anti–PD‐1: DMSO (s.c.) + anti–PD‐1 antibody; CCR6 Antagonist + Isotype: CCR6 antagonist (s.c.) + IgG2a isotype control; CCR6 Antagonist + anti–PD‐1: CCR6 antagonist (s.c.) + anti–PD‐1 antibody. Drug administration:anti–PD‐1 antibody (Clone RMP1‐14; MCE, #HY‐P99144) or Isotype IgG2a (Clone 12B9; Syd Labs, #PA007129) was administered intraperitoneally at a dose of 200 µg per mouse, twice a week. The CCR6 antagonist HY‐151435 was administered subcutaneously at 1 mg/kg once daily as a literature‐informed proof‐of‐concept regimen. This dose and route were selected based on prior in vivo studies showing biological activity of CCR6 antagonism at the 1 mg/kg dose level and published subcutaneous use of HY‐151435 at 1–2 mg/kg in mice [38, 39]. Tumor progression was monitored every three days using digital calipers. Tumor volume was calculated using the formula: V = 0.5×Length×Width×Width.

### 5.11 Flow Cytometry

Tumor tissues were harvested, mechanically minced into small pieces, and enzymatically digested in DMEM containing 2 mg/mL Dispase II (Roche, #4942078001), 2 mg/mL Collagenase I (Sigma, #C0130), and 0.1 mg/mL Hyaluronidase (Sigma, #H3506). Digestion was performed at 37°C for 30–60 min with gentle agitation. The resulting cell suspensions were filtered through a 100‐µm cell strainer and resuspended in phosphate‐buffered saline (PBS). Red blood cells were removed using RBC Lysis Buffer (BioLegend, #420302) by incubation on ice for 10 min. Single‐cell suspensions were washed with PBS and first stained with the Zombie Red Fixable Viability Kit (BioLegend, #423110; 1:1000 dilution) for 20 min at room temperature to exclude dead cells. To prevent non‐specific binding, cells were blocked with anti‐mouse CD16/32 Fc block antibody (BioLegend, #101320) for 10 min on ice. For surface marker profiling, cells were incubated with fluorochrome‐conjugated primary antibodies in Cell Staining Buffer (BioLegend, #420201) for 30 min at 4°C in the dark. The following surface antibodies were used: CD45, CD3ε, CD4, CD8a, CD11b, F4/80, and CD86. All antibodies were purchased from BioLegend unless otherwise stated. For intracellular staining, cells were stimulated with Cell Activation Cocktail containing Brefeldin A, fixed, permeabilized, and stained with antibodies against Granzyme B, PRF1, CD206. Detailed information on all antibodies, including clone, supplier, catalog number, dilution or amount used, and application, is provided in Supplementary Table S3. Flow cytometry data were acquired on a CytoFLEX S flow cytometer (Beckman Coulter). Data analysis was performed using FlowJo software (version 10; TreeStar, Ashland, OR).

### 5.12 Immunoprecipitation and Mass Spectrometry

To identify CHST1‐interacting proteins, HEK‐293T cells were transiently transfected with Flag‐tagged CHST1 vectors. 48 h post‐transfection, cells were harvested and washed twice with cold PBS. Immunoprecipitation was performed using an IP Kit (Beyotime, #P2175) according to the manufacturer's instructions. Briefly, cells were lysed in cold NP‐40 lysis buffer supplemented with PMSF and protease inhibitors. The lysates were centrifuged, and the supernatants were incubated overnight at 4°C with anti‐Flag antibodies or control IgG conjugated to protein A/G beads. The immunoprecipitated complexes were eluted and subjected to liquid chromatography‐tandem mass spectrometry (LC‐MS/MS) analysis. For validation via Co‐IP, the eluted protein complexes were separated by SDS‐PAGE and analyzed by Western blotting.

### 5.13 Nuclear‐Cytoplasmic Fractionation

Subcellular fractionation was performed to isolate nuclear and cytoplasmic protein pools using a Nuclear and Cytoplasmic Protein Extraction Kit (Epizyme, #PC204) following the manufacturer's protocol. The purity of the cytoplasmic and nuclear fractions was verified by Western blotting using antibodies against GAPDH (cytoplasmic marker; 1:5000, Epizyme, #LF205S) and Histone H3 (nuclear marker; 1:1000, Epizyme, #R013738), respectively.

### 5.14 Structural Prediction

The CHST1–NKRF complex structure was predicted using AlphaFold3. Full‐length murine Chst1 and Nkrf protein sequences were used as input for complex prediction. The prediction was performed using the AlphaFold3 server with default settings. No manually assigned structural templates, ligands, nucleic acids, post‐translational modifications, or experimental restraints were included. Five ranked models were generated by AlphaFold3, and the top‐ranked model was selected for visualization and downstream interpretation based on the model ranking and confidence metrics provided by AlphaFold3. Model confidence was evaluated using predicted local distance difference test scores and predicted aligned error plots.

Structural visualization and interface inspection were performed in PyMOL. The predicted CHST1–NKRF interaction interface was examined across the ranked models, with particular attention to CHST1 residues located in spatial proximity to the predicted nuclear localization sequence region of NKRF. Candidate interface residues were prioritized based on their recurrence across models, spatial proximity to the NKRF nuclear localization sequence, and local model confidence. The AlphaFold3 prediction was used as a hypothesis‐generating analysis to guide subsequent interpretation and design of CHST1 interface mutants, rather than as definitive structural evidence of direct binding.

### 5.15 Combined Signature Construction and Validation

To evaluate the translational relevance of the CHST1–NKRF–CCL20 axis, we constructed a mechanism‐informed composite expression signature based on CHST1, NKRF, and CCL20. Gene expression data were extracted from the external I‐SPY2 early TNBC cohort (GSE194040) and from the internal FASCINATE‐N TNBC cohort. For each cohort, expression values of the three genes were Z‐score normalized across samples before signature calculation. Because high CHST1 and CCL20 expression were associated with immunosuppressive remodeling and poor therapeutic response, whereas NKRF was considered a negative regulator of NF‐κB‐associated CCL20 transcription, a composite response‐oriented score was calculated using the following predefined formula: Response score = −1.5 × Z(CHST1) + 0.5 × Z(NKRF) − 0.5 × Z(CCL20). The coefficients were defined a priori based on the proposed biological direction of the CHST1–NKRF–CCL20 axis and were kept fixed across all cohorts and treatment arms without cohort‐specific re‐training. Pathological complete response was coded as a binary outcome (pCR = 1, non‐pCR = 0). Logistic regression was used to model the association between the composite score and pCR status and to estimate the predicted probability of pCR for each sample. In the I‐SPY2 cohort, the predictive performance of the composite score was evaluated separately in the chemotherapy‐only and chemoimmunotherapy arms using precision–recall (PR) curve analysis. In the FASCINATE‐N cohort, predictive performance was assessed using both receiver operating characteristic (ROC) and PR curve analyses. ROC AUC values were calculated using the pROC package, and area under the precision–recall curve (PR AUC) values were calculated using the PRROC package. For visualization, patients were stratified into high‐ and low‐score groups according to the median composite score within each cohort or treatment arm. All analyses were performed in R (version 4.3.2) using dplyr, ggplot2, pROC, PRROC, ggpubr, RColorBrewer, and openxlsx.

### 5.16 Quantification and Statistical Analysis

Statistical analyses were performed using GraphPad Prism software (version 8.0.0) and R statistical software (version 4.3.2). To determine statistical significance, the normality of data distribution was first assessed. For comparisons between two groups, the unpaired Student's *t*‐test was used for normally distributed data, while the Mann‐Whitney *U* test was applied for non‐parametric data. For comparisons among three or more groups, one‐way analysis of variance (ANOVA) followed by Tukey's post‐hoc test was utilized. Non‐parametric data involving three or more groups were analyzed using the Kruskal‐Wallis test followed by Dunn's post‐hoc test. Categorical variables were compared using the Chi‐square test. Correlation analyses were performed using Pearson's (for normal distribution) or Spearman's (for non‐normal distribution) correlation coefficients. A two‐sided *p*‐value of < 0.05 was considered statistically significant. Exact sample sizes (n) and statistical parameters are specified in the corresponding figure legends.

## Author Contributions

J.J.L., Z.M.S., and L.C. conceived the study, supervised the project, and designed the methodologies. J.J.L. and Z.M.S. provided experimental guidance. S.H.J. and X.H.Y. performed the experiments. S.H.J., X.H.Y., and R.Q.W. conducted the bioinformatics analysis, prepared the figures, and analyzed the data. S.H.J., X.H.Y., R.Q.W., and Q.Z. wrote the manuscript and participated in the revision. All authors have read and agreed to the published version of the manuscript.

## Conflicts of Interest

The authors declare no conflicts of interest.

## Supporting information




**Supporting File**: advs77356‐sup‐0001‐SuppMat.docx.

## Data Availability

The results shown here are in part based upon data generated by The Cancer Genome Atlas (TCGA) Research Network, available at the GDC Data Portal (https://portal.gdc.cancer.gov/), and the Molecular Taxonomy of Breast Cancer International Consortium (METABRIC) dataset, accessed via cBioPortal (https://www.cbioportal.org/study/summary?id = brca_metabric). The microarray and sequence data of the FUSCC TNBC cohort have been deposited in the NCBI Gene Expression Omnibus (GEO) under accession number GSE118527 and the Sequence Read Archive (SRA) under accession number SRP157974. Public datasets used for independent validation in this study include the I‐SPY2 TNBC immunotherapy cohort (GSE194040), the METABRIC cohort. This paper does not report original code. Any additional information required to reanalyze the data reported in this paper is available from the corresponding author upon request.
