# Supplementary material for: CHST1 Drives Immunotherapy Resistance in Triple‐Negative Breast Cancer by Orchestrating an Immunosuppressive Microenvironment via the NKRF–CCL20–Macrophage Axis
Source: Adv Sci (Weinh). 2026 Aug 24;13(63):e77356. doi: 10.1002/advs.77356 (PMC13502349; doi:10.1002/advs.77356)
Supplement: Supplementary file 1 [file ADVS-13-e77356-s001.docx]

**Supporting Information**

**CHST1 Drives Immunotherapy Resistance in Triple-Negative Breast Cancer by Orchestrating an Immunosuppressive Microenvironment via the NKRF–CCL20–Macrophage Axis**

Shu-Hao Jiang^†^, Xiao-Han Ying^†^, Ruo-Qing Wang^†^, Qun Zhang, Li Chen, Zhi-Ming Shao*, and Jun-Jie Li*

**Figure S1. CHST1 expression is elevated in TNBC tumor tissues compared with paired adjacent normal tissues.**

A, CHST1 mRNA expression in paired tumor and adjacent normal tissues from the FUSCC TNBC cohort (n = 88 pairs). Each line connects matched tumor and normal samples from the same patient.

**Figure S2. Baseline CHST1/Chst1 expression, genetic model validation, and cell-autonomous proliferation assays in TNBC models.**

A,B, Baseline CHST1 protein (A) and mRNA (B) expression in human TNBC cell lines. VINCULIN served as the loading control for Western blotting.

C,D, Baseline Chst1 protein (C) and mRNA (D) expression in murine mammary tumor cell lines. VINCULIN served as the loading control for Western blotting.

E,F, Validation of CHST1 depletion in MDA-MB-468 cells expressing non-targeting sgRNA control or two independent CHST1-targeting sgRNAs by Western blotting (E) and RT-qPCR (F).

G,H, Validation of CHST1 overexpression in MDA-MB-231 cells expressing vector control or CHST1 by Western blotting (G) and RT-qPCR (H).z

I,J, Validation of Chst1 knockdown in 4T1 cells expressing shChst1 NC or two independent Chst1-targeting shRNAs by Western blotting (I) and RT-qPCR(J). GAPDH served as the loading control.

K,L, Validation of Chst1 overexpression in AT3 cells expressing vector control or Chst1 by Western blotting (K) and RT-qPCR(L). VINCULIN served as the loading control.

M–P, Representative colony formation images of MDA-MB-468 (M), MDA-MB-231 (N), 4T1 (O), and AT3 (P) cells following CHST1/Chst1 depletion or overexpression.

Q–T, Quantification of colony formation assays in MDA-MB-468 (Q), MDA-MB-231 (R), 4T1 (S), and AT3 (T) cells.

U–X, CCK-8 assays showing the proliferation of MDA-MB-468 (U), MDA-MB-231 (V), 4T1 (W), and AT3 (X) cells following CHST1/Chst1 depletion or overexpression.

Data are presented as mean ± SEM. Statistical significance was determined as described in the Methods. ns, not significant; **P < 0.01; ***P < 0.001.

**Figure S3. CHST1 modulation does not markedly alter TNBC cell migration or invasion in vitro, and exploratory correlations with immune-related markers in the discovery cohort.**

A–C, Transwell migration and Matrigel invasion assays in MDA-MB-468 cells expressing non-targeting sgRNA control or two independent CHST1-targeting sgRNAs. Representative images are shown in A. Quantification of migrated (B) and invaded (C) cells showed no significant differences among the indicated groups.

D–F, Transwell migration and Matrigel invasion assays in MDA-MB-231 cells expressing vector control or CHST1. Representative images are shown in D. Quantification of migrated (E) and invaded (F) cells showed no significant differences between the indicated groups.

G–J, Scatter plots showing exploratory correlations between CHST1 mRNA expression and immune-related genes in the FASCINATE-N discovery cohort (n = 11), including CD274 (PD-L1) (G), CTLA4 (H), CD8A (I), and GZMB (J). Correlation coefficients and P values are shown in each panel.

Data are presented as mean ± SEM. Statistical significance was determined as described in the Methods. ns, not significant; *P < 0.05; **P < 0.01; ***P < 0.001; ****P < 0.0001.

**Figure S4. Single-cell distribution of CHST1 and its association with macrophage remodeling in vitro.**

A,B, UMAP visualization of public breast cancer single-cell RNA-seq data showing annotated major cell populations (A) and CHST1 expression across cell populations within the tumor microenvironment (B).

C,D, RT-qPCR analysis of macrophage polarization markers in RAW264.7 cells after non-contact co-culture with 4T1 cells expressing shChst1 NC or two independent Chst1-targeting shRNAs (C), or with AT3 cells expressing vector control or Chst1 (D). M1-like markers included Cd86, Tnf-α, and Inos, whereas M2-like markers included Arg2, Cd206, and Il10.

E,F, Flow cytometry analysis of CD206 expression in RAW264.7 cells from the 4T1 co-culture system. Representative histograms are shown in E, and the percentage of CD206⁺ RAW264.7 cells is quantified in F.

G,H, Flow cytometry analysis of CD206 expression in RAW264.7 cells from the AT3 co-culture system. Representative histograms are shown in G, and the percentage of CD206⁺ RAW264.7 cells is quantified in H.

Data are presented as mean ± SEM. Statistical significance was determined as described in the Methods. ns, not significant; *P < 0.05; **P < 0.01; ***P < 0.001; ****P < 0.0001.

**Figure S5. Validation of Chst1 mutant expression and Ccl20-dependent macrophage remodeling in murine TNBC models.**

A, Western blot analysis of Flag-tagged Chst1-WT and Chst1-R243Q expression and immunoprecipitation in AT3 cells. Flag-tagged proteins were enriched by anti-Flag immunoprecipitation, and whole-cell lysates were analyzed as input controls. GAPDH served as the loading control.

B, RT-qPCR analysis of macrophage polarization markers in RAW264.7 cells co-cultured with AT3 cells expressing vector control, Flag-Chst1, or Flag-Chst1 with Ccl20 knockdown. M1-like markers included Cd86, Tnf-α, and Inos, whereas M2-like markers included Arg2, Cd206, and Il10.

Data are presented as mean ± SEM. Statistical significance was determined by one-way ANOVA followed by multiple-comparison test. ns, not significant; **P < 0.01; ***P < 0.001; ****P < 0.0001.

**Figure S6. Chst1 manipulation does not alter total NKRF abundance and working model of the CHST1–NKRF–CCL20 axis.**

A, RT-qPCR analysis of Nkrf mRNA expression in 4T1 cells expressing shChst1 NC or two independent Chst1-targeting shRNAs.

B, Western blot analysis of total Nkrf and Chst1 protein levels in whole-cell lysates from 4T1-shChst1 NC, 4T1-shChst1#1, and 4T1-shChst1#2 cells.

C, RT-qPCR analysis of Nkrf mRNA expression in AT3 cells expressing vector control or Flag-Chst1.

D, Western blot analysis of total Nkrf and Flag-Chst1 protein levels in whole-cell lysates from AT3-vector and AT3-Flag-Chst1 cells.

E, Proposed working model of the CHST1–NKRF–CCL20–macrophage regulatory program. In CHST1-high tumors, CHST1 association with NKRF is linked to increased NKRF cytoplasmic retention and reduced nuclear accumulation. This localization change is proposed to weaken NKRF-mediated repression of an NF-κB-associated CCL20 transcriptional program. Increased tumor-cell-derived CCL20 may subsequently contribute to macrophage recruitment and M2-like remodeling.

**Figure S7. Additional validation of Chst1 overexpression and CCR6 blockade combined with anti–PD-1 therapy in syngeneic TNBC models.**

A, Western blot analysis confirming Flag-Chst1 overexpression in 66cl4 cells.

B–D, AT3-Chst1-NC tumors were established in syngeneic mice and treated with Vehicle (DMSO) plus IgG2a isotype control, Vehicle (DMSO) plus anti–PD-1, CCR6 antagonist plus IgG2a isotype control, or CCR6 antagonist plus anti–PD-1. Representative endpoint tumors are shown in B. Tumor growth curves (C) and endpoint tumor weights (D) were measured.

E–G, AT3-Chst1-OE tumors were established in syngeneic mice and treated as described above. Representative endpoint tumors are shown in E. Tumor growth curves (F) and endpoint tumor weights (G) were measured.

H,I, Flow cytometry analysis of Granzyme B expression in tumor-infiltrating CD8⁺ T cells from endpoint AT3 tumors under the indicated treatment conditions. Representative histograms are shown in H, and the percentage of Granzyme B⁺ CD8⁺ T cells is quantified in I.

Data are presented as mean ± SEM. Statistical significance was determined as described in the Methods. ns, not significant; *P < 0.05; **P < 0.01; ***P < 0.001; ****P < 0.0001.

**Figure S8. Raw immunoblot images corresponding to the immunoblots presented in the manuscript.**

Raw and unedited immunoblot images corresponding to Figure 5D (A), Figure 5E (B), Figure 5F (C), Figure 5G (D), Figure 5K (E), Figure 5L (F), Figure S2A (G), Figure S2C (H), Figure S2E (I), Figure S2G (J), Figure S2I (K), Figure S2K (L), Figure S5A (M), Figure S6B (N), Figure S6D (O), and Figure S7A (P) are shown. Each raw immunoblot image is labeled with its corresponding figure/panel and target protein to facilitate direct traceability to the immunoblot data presented in the manuscript.

**Table S1. Clinical characteristics of the 11 TNBC patients in the discovery cohort.**

| ID | Age (years) | TNM Stage | Neoadjuvant Treatment | Outcome |
| --- | --- | --- | --- | --- |
| P001 | 53 | T2N2 | Chemotherapy + Immunotherapy | non-pCR |
| P002 | 44 | T2N2 | Chemotherapy + Immunotherapy | pCR |
| P003 | 69 | T1N2 | Chemotherapy + Immunotherapy | non-pCR |
| P004 | 44 | T2N0 | Chemotherapy + Immunotherapy | non-pCR |
| P005 | 24 | T3N1 | Chemotherapy + Immunotherapy | pCR |
| P006 | 56 | T3N1 | Chemotherapy + Immunotherapy | non-pCR |
| P007 | 41 | T3N2 | Chemotherapy + Immunotherapy | non-pCR |
| P008 | 33 | T2N2 | Chemotherapy + Immunotherapy | pCR |
| P009 | 33 | T2N2 | Chemotherapy + Immunotherapy | pCR |
| P010 | 53 | T4N2 | Chemotherapy + Immunotherapy | pCR |
| P011 | 33 | T2N2 | Chemotherapy + Immunotherapy | pCR |

Patient demographics, TNM staging, neoadjuvant treatment regimens, and therapeutic responses are listed. All patients were treated with a combination of chemotherapy and immunotherapy. pCR, pathological complete response; non-pCR, non-pathological complete response.

**Table S2. List of primer sequences used in this study.**

| Primer name | Primer sequence (5’ to 3’) |
| --- | --- |
| mCHST1-F | CCCCAGGATGATAGCGTCAG |
| mCHST1-R | ACACAGCAGTTACCTTCCCT |
| hCHST1-F | GACTTTCTCCCCAGCTGCAT |
| hCHST1-R | CCAAGGGGTGAGGTCAAAGA |
| mTnfa-F | CCCTCACACTCAGATCATCTTCT |
| mTnfa-R | GCTACGACGTGGGCTACAG |
| mCd86-F | TGTTTCCGTGGAGACGCAAG |
| mCd86-R | TTGAGCCTTTGTAAATGGGCA |
| mInos-F | GTTCTCAGCCCAACAATACAAGA |
| mInos-R | GTGGACGGGTCGATGTCAC |
| mArg2-F | TCCTCCACGGGCAAATTCC |
| mArg2-R | GCTGGACCATATTCCACTCCTA |
| mCD206-F | CTCTGTTCAGCTATTGGACGC |
| mCD206-R | CGGAATTTCTGGGATTCAGCTTC |
| mIl10-F | GCTCTTACTGACTGGCATGAG |
| mIl10-R | CGCAGCTCTAGGAGCATGTG |
| hGAPDH-F | CACCAGGGCTGCTTTTAACTC |
| hGAPDH-R | GACAAGCTTCCCGTTCTCAG |
| hCD86-F | GGGCTTTTTATCTTCACCTTTC |
| hCD86-R | GTTGCGAGGCCGCTTCT |
| hiNOS-F | GGCTGCCAAGCTGAAATGA |
| hiNOS-R | TCCTTCTTCGCCTCGTAAGG |
| hCD163-F | GTGCAGAAAACCCACAAAAA |
| hCD163-R | CGAAATGGCCAACAGAAACA |
| hCD206-F | GCTACTGGAAGACAGCACATTGC |
| hCD206-R | GGAATCCATGCTGTGTGATCTG |
| hArg1-F | CAGTTTGGCAATTGGAAGCA |
| hArg1-R | CACTTGTGGTTGTCAGTGGAAGTG |

The specific nucleotide sequences (5' to 3') for all human (h) and murine (m) primers utilized in RT-qPCR assays are listed. F, forward primer; R, reverse primer.

**Table S3. Antibodies used in this study.**

| Target | Host species | Clone | Supplier | Catalog No. | Dilution / amount | Application |
| --- | --- | --- | --- | --- | --- | --- |
| CHST1 | Mouse | A-2 | Santa Cruz Biotechnology | sc-365021 | 1:1000 | Western blot |
| NKRF | Rabbit | Polyclonal | Proteintech | 14693-1-AP | 1:5000 | Western blot |
| Histone H3 | Rabbit | 42I15C31 | Epizyme | R013738 | 1:1000 | Western blot |
| GAPDH | Mouse | 1E6D9 | Proteintech | 60004-1-Ig | 1:100000 | Western blot |
| HRP-conjugated anti-mouse IgG | Goat | Polyclonal | Epizyme | LF101 | 1:1000 | Western blot |
| HRP-conjugated anti-rabbit IgG | Goat | Polyclonal | Thermo Fisher | 31460 | 1:100000 | Western blot |
| Zombie Red Fixable Viability Kit | — | — | BioLegend | 423110 | 1:1000 | Flow cytometry |
| CD16/32 Fc block | Rat | 93 | BioLegend | 101320 | 1 μg/10⁶ cells in 100 μL | Flow cytometry |
| CD45 | Rat | 30-F11 | BD Pharmingen | 557659 | 0.06 μg/test | Flow cytometry |
| CD3ε | Hamster | 145-2C11 | BioLegend | 100327 | 0.25 µg/10^6^ cells in 100 µl | Flow cytometry |
| CD4 | Rat | RM4-5 | BioLegend | 100536 | 0.25 µg/10^6^ cells in 100 µl | Flow cytometry |
| CD8a | Rat | 53-6.7 | BioLegend | 100706 | 1.0 µg/10^6^ cells in 100 µl | Flow cytometry |
| CD11b | Rat | M1/70 | BioLegend | 101206 | 0.25 µg/10^6^ cells in 100 µl | Flow cytometry |
| F4/80 | Rat | BM8 | BioLegend | 123128 | 1.0 µg /10^6^ cells in 100 µl | Flow cytometry |
| CD86 | Rat | GL-1 | BioLegend | 105008 | 1.0 µg /10^6^ cells | Flow cytometry |
| Granzyme B (GZMB) | Mouse | QA16A02 | BioLegend | 372208 | 5 µl/10^6^ cells in 100 µl | Flow cytometry |
| Perforin (PRF1) | Rat | S16009B | BioLegend | 154404 | 0.5 µg/10^6^ cells in 100 µl | Flow cytometry |
| CD206 | Rat | C068C2 | BioLegend | 141708 | 0.5 µg/10^6^ cells in 100 µl | Flow cytometry |

Abbreviations: HRP, horseradish peroxidase.
